# Neurogenic Processes Are Induced by Very Short Periods of Voluntary Wheel-Running in Male Mice

**DOI:** 10.3389/fnins.2017.00385

**Published:** 2017-07-13

**Authors:** Teresa Gremmelspacher, Johannes Gerlach, Alix Hubbe, Carola A. Haas, Ute Häussler

**Affiliations:** ^1^Experimental Epilepsy Research, Department of Neurosurgery, Faculty of Medicine, Medical Center-University of Freiburg Freiburg, Germany; ^2^BrainLinks-BrainTools, Cluster of Excellence, University of Freiburg Freiburg, Germany; ^3^Faculty of Biology, University of Freiburg Freiburg, Germany

**Keywords:** neurogenic niche, neurogenesis, proliferation, hippocampus, progenitor cells, voluntary wheel running, BDNF

## Abstract

Even in the adult mammalian brain progenitor cells proliferate and give rise to young neurons which integrate into the neuronal network. The dentate gyrus possesses such a neurogenic niche reactive to external stimuli like physical activity. In most studies mice or rats have been exposed to wheel running for periods of several weeks to activate neurogenesis while early neurogenic processes induced by very short running periods are less well understood. To address this issue, we allowed male C57Bl/6 mice free access to a running wheel for 2 or 7 days. We injected bromodeoxyuridine (BrdU) before the last running night, respectively, and quantified cell proliferation with immunocytochemistry for BrdU and Ki-67. Furthermore, we performed immunocytochemistry for doublecortin (DCX) and real-time RT-qPCR for NeuroD1 to characterize and quantify changes in neurogenesis on the protein and mRNA level. Real-time RT-qPCR for neurogenic niche factors (BDNF, FGF-2, BMP4, Noggin) was used to detect changes in the molecular composition of the neurogenic niche. Interestingly, we observed that cell proliferation was already affected after 2 days of running showing a transient decrease, which was followed by a rebound with increased proliferation after 7 days. Neurogenesis was stimulated after 2 days of running, reflected by elevated NeuroD1 mRNA levels, and it was significantly increased after 7 days as indicated by DCX immunostaining. On the level of niche factors we observed changes in expression in favor of neuronal differentiation (increased BDNF mRNA expression) and proliferation (decreased BMP4 mRNA expression) already after 2 days, although increased proliferation is reflected on the cellular level only later. In summary, our data show that 2 days of running are sufficient to activate neurogenic processes and we hypothesize that a strong pressure toward differentiation privileges neurogenesis while proliferation lags behind.

## Introduction

Neurogenesis is a continuous process occurring in distinct areas of the brain from before birth until adulthood. In the subgranular zone (SGZ) of the dentate gyrus new granule cells develop from glia-like stem cells via different precursor stages in a fragile process in which the microenvironment of the neurogenic niche acts as a link between external influences and the fate of progenitor maturation. A well-investigated external stimulus is physical activity which increases neurogenesis, improves spatial learning and augments long-term potentiation in the dentate gyrus (e.g., van Praag et al., 1999b,a).

Proliferation and fate decision of progenitor cells in the neurogenic niche depend on various niche factors. Among these, bone morphogenetic protein 4 (BMP4) is synthesized in the SGZ (Xu et al., 2013) and modulates whether stem cells enter the cell cycle: Blocking BMPs induces proliferation and differentiation of progenitors in the SGZ (Bonaguidi et al., 2008) while overexpression leads to reduced stem cell/progenitor numbers and, consequently, reduced neurogenesis (Gobeske et al., 2009). In contrast, the BMP inhibitor Noggin, which is expressed by ependymal cells (Lim et al., 2000), elevates cell proliferation in the SGZ, mainly by increasing proliferative activity of quiescent type I stem cells (Bonaguidi et al., 2008), the cell type which is also activated by voluntary wheel running (Lugert et al., 2010). Asymmetric cell division then leads to a rise of the transiently amplifying population of type II cells and as consequence to an increase in neurogenesis. In fact, a decrease in BMP4 together with an increase in Noggin expression was suggested to be necessary for running-induced progenitor proliferation (Gobeske et al., 2009).

While BMP and Noggin mainly affect proliferation, fibroblast growth factor-2 (FGF-2), which is expressed by granule cells and astrocytes in the dentate gyrus (Araujo and Cotman, 1992; Zechel et al., 2010) acts on differentiation and is responsible for the neurogenic fate of proliferating cells. Consequently, FGF-2 knockout mice have reduced numbers of NeuroD1- and doublecortin (DCX)-positive neuronal precursors (Werner et al., 2011) without changes in the number of proliferating cells. Brain-derived neurotrophic factor (BDNF) influences proliferation, differentiation and neuronal protection (Vivar et al., 2013) and is secreted in an activity-dependent manner by neurons and astrocytes. After physical activity the expression of FGF-2 and BDNF is elevated, resulting in increased neurogenesis (Neeper et al., 1996; Gomez-Pinilla et al., 1997).

Studies on running-induced changes in the expression of neurogenic niche factors have mostly been performed in rats that had run for a few days to several weeks (Neeper et al., 1996; Gomez-Pinilla et al., 1997; Vaynman et al., 2003), yet the effects on proliferation and neurogenesis have rarely been investigated in parallel. In contrast, studies focusing on the latter often used running periods of several weeks in mice or rats and only a few focused on short term running. We conducted a comprehensive study with C57Bl/6 mice which ran for short periods and analyzed mRNA expression patterns of niche factors (BDNF, FGF-2, BMP4, Noggin), as well as expression of proliferation [Ki-67, bromodeoryuridine (BrdU)] and neurogenesis markers (DCX and NeuroD1).

## Materials and methods

### Animals, running wheel paradigm and BrdU injections

All animal procedures were carried out in accordance with the guidelines of the European Community's Council Directive of 22 September 2010 (2010/63/EU) and approved by the regional council (Regierungspräsidium Freiburg).

Adult (8–12 weeks-old) male C57Bl/6N mice (Charles River, Sulzfeld, Germany) were acclimatized to the experiment room for 24 h before being transferred in pairs in cages with free access to running wheels (TSE Systems, Bad Homburg, Germany) or in standard cages [sedentary (SDN) mice] with a 12/12 h light/dark cycle, at room temperature (RT, 22 ± 2°C) with food and water *ad libitum*. In a preliminary study mice were allowed to use the running wheels for 10 days. To analyze the effect of short-term running, mice were randomly arranged in two groups which ran either two [2 day runners (2DR)] or 7 days [7 day runners (7DR)] before sacrifice. Running data (wheel rotations and usage duration) were measured with Phenomaster Software (TSE Systems).

To monitor cell proliferation, a subset of mice was injected with bromodeoxyuridine (BrdU) (i.p., 50 mg/kg body weight in 0.9% saline; Sigma-Aldrich, Taufkirchen, Germany) shortly before 7 p.m., the time point when light was switched off, corresponding to the onset of the running period. BrdU was applied at the beginning of the last running night for 2DR and 7DR groups, respectively, and the night before perfusion for SDN mice.

### Perfusion and tissue preparation

The next morning, mice were deeply anesthetized (200 mg/kg ketamine hydrochloride, 10 mg/kg xylazine, 0.2 mg/kg acetylpromazine) and perfused intracardially with 0.9% NaCl, followed by 4% paraformaldehyde [PFA, in 0.1 M phosphate buffer (PB), pH 7.4, 4 min]. Brains were excised and post-fixed for 4 h in 4% PFA.

For immunocytochemistry vibratome sections (coronal plane, 50 μm; Leica, VT1000S, Bensheim, Germany) were distributed in series of five and collected in PB. For *in situ* hybridization (ISH) brains were cryoprotected in 30% sucrose, frozen in isopentane, sliced on a cryostat (coronal plane, 50 μm) and sections were collected in RNAse-free 2x SSC (1x SSC = 0.15 M NaCl, 0.015 M sodium citrate, pH 7.0).

For real-time RT-qPCR analysis mice were decapitated under isoflurane anesthesia, hippocampi were freshly prepared under RNAse-free conditions and frozen in RNA-later (Qiagen, Stockach, Germany).

### Immunocytochemistry

All sections were treated equally using a standard free-floating protocol. For BrdU detection, sections were pretreated in 2 N HCl (30 min, 37°C) and neutralized in 0.1 M Tris-buffered saline (pH 8.5, 10 min). Preincubation with 0.25% Triton X-100 and 10% normal serum for 30 min was followed by incubation with the primary antibody (4 h, RT + overnight, 4°C). The following primary antibodies were applied in consecutive sections to avoid overlap in quantification for either antibody: Goat polyclonal anti-DCX (1:500, sc8066, Santa Cruz Biotechnology Inc., Dallas, TX, USA), mouse monoclonal anti-Ki-67 (1:100, NCL-L-Ki67-MM1, Leica Biosystems Newcastle Ltd., Newcastle Upon Tyne, UK) or rabbit polyclonal anti-Ki-67 (1:500, ab15580, Abcam, Cambridge, UK) and rat monoclonal anti-BrdU (1:500, OBT0030, AbD Serotec, Oxford, UK).

A fluorescent secondary rabbit anti-goat antibody with Cy3-labeling was used (1:400; Jackson ImmunoResearch Laboratories Inc, West Grove, PA, USA) together with DAPI (4′,6-diamino-2-phenylindole, 1:10,000) for counterstaining and sections were coverslipped with anti-fading medium (Immu-Mount, Thermo Shandon, Dreieich, Germany).

For counting of BrdU- and Ki-67-positive cells, immunoperoxidase detection was performed using a biotinylated rabbit anti-rat (1:250), biotinylated rabbit anti-mouse (1:250) or biotinylated goat anti-rabbit (1:250) antibody (Vector Laboratories, Burlingame, CA, USA) and the avidin-biotin complex (Vectastain Elite Kit, Vector Laboratories). Detection was achieved with 0.05% 3′,3′-diaminobenzidine tetrahydrochloride (DAB, Sigma-Aldrich) and 0.002% H_2_O_2_. Sections were mounted, dehydrated in ethanol, cleared in xylene and coverslipped with hypermount (Thermo Shandon).

### *In situ* hybridization

Doublecortin mRNA expression was localized with digoxigenin (DIG)-labeled cDNA probes generated by *in vitro* transcription as described earlier (Heinrich et al., 2006). After pretreatment in hybridization buffer (50% formamide, 4x SSC, 50 mM NaH_2_PO_4_, 250 μg/ml heat-denatured salmon sperm DNA, 100 μg/ml tRNA, 5% dextransulfate and 1% Denhardt's solution) diluted with 2x SSC (1:1) for 15 min, cryostat sections were prehybridized in the same buffer for 60 min at 55°C. For hybridization, DIG-labeled DCX anti-sense or sense cRNA probes (100 ng/ml) were added and incubated at 55°C overnight. Washing in 2x SSC (2 × 15 min, RT), 2x SSC and 50% formamide (15 min, 65°C), 0.1x SSC and 50% formamide (15 min, 65°C) and Tris-buffered saline (2 × 10 min, RT) was followed by incubation in blocking buffer (1% blocking reagent in Tris-buffered saline, 60 min, RT). The DIG-labeled hybrids were detected by immunocytochemistry with an anti-DIG antibody conjugated to alkaline phosphatase (1:1500, sheep, Roche, Mannheim, Germany) which reduced nitroblue tetrazolium under the addition of 5-Brom-4-chlor-3-indoxylphsophate (BCIP). Sections were coverslipped with Kaiser's glycerol gelatine.

### RNA extraction, reverse transcription and quantitative real-time PCR

The hippocampus was homogenized and total RNA was extracted and purified using the RNeasy Mini Kit (Qiagen, Hilden, Germany) according to the manufacturer's instructions with dithiothreitol (DTT) as reducing agent. Reverse transcription was performed with 1 μg RNA using the Maxima First Strand cDNA Synthesis Kit (Fermentas, Life Technologies GmBH, Darmstadt, Germany) based on random hexamer and oligo(dT)_18_ priming. Quantification of mRNA expression was performed by real-time RT-qPCR on an iQ™5 Real-Time PCR Detection System (Bio-Rad Laboratories, Munich, Germany) in the presence of SYBR Green (Applied Biosystems, Darmstadt, Germany).

The following mouse-specific primer sequences were used: BDNF: forward: 5′-GCTCACACTCCACTGCCCAT-3′, reverse: 5′-TCCCTGACCCATGCCAGAAGA-3′ (product length: 71 bp); BMP4: forward: 5′-CGAGCCAACACTGTGAGGAGTTTC-3′, reverse: 5′-TTCTCTGGGATGCTGCTGAGGTT-3′ (product length: 113 bp); Noggin: forward: 5′-TGACCTAGGCAGCCGCTTTT-3′, reverse: 5′-TGGCTTACACACCATGCCCTC-3′ (product length: 97 bp); FGF-2: forward: 5′-GAAGAGCGACCCACACGTCAAA-3′, reverse: 5′-CAAGGTACCGGTTGGCACACA-3′ (product length: 89 bp), NeuroD1: forward: 5′-CCGAGGCTCCAGGGTTATGAGA-3′, reverse: 5′-CCCGCTCTCGCTGTATGATTTGG-3′ (product length 90 bp); housekeeping gene S12: forward: 5′-GGCATAGCTGCTGGAGGTGTAA-3′, reverse: 5′-GGGCTTGGCGCTTGTCTAA-3′ (product length: 129 bp). All samples were measured in duplicates. To control for specificity of the amplification reaction, melting curves were analyzed and the size of amplification products was verified by agarose gel electrophoresis.

The calculation of relative mRNA expression values was performed according to the Cy0 method (Guescini et al., 2008). The Ct values of each target gene were normalized to the ribosomal protein S12 (expressed as Δ Ct) and relative expression was calculated by 2^(−ΔCt)^.

### Microscopy and image analysis

Microscopy was performed with an AxioImager 2 (ZEN software; Zeiss, Göttingen, Germany). Images were taken with a digital camera (AxioCam MRm for fluorescence, AxioCam MRc5 for bright field, both Zeiss), a 10-fold objective (Plan Apochromat, NA 0.45); identical exposure times were used for all sections that were compared. Images were analyzed with ImageJ software (Version 1.50, National Institutes of Health (NIH), USA).

For the preliminary analysis of differences in running-induced neurogenesis along the septotemporal axis of the hippocampus sections from different levels (6 to 10 intact sections per mouse) were localized according to Franklin and Paxinos (2008). For densitometric analysis of DCX labeling, all images were transferred to gray scale and identical contrast enhancement was performed. Background staining was measured for each image in CA1 *stratum radiatum* and subtracted from the respective whole image. Subsequently, the dentate gyrus but sparing the hilus was selected as region of interest (ROI). Mean gray value and standard deviation were measured for each section of SDN mice and average mean + standard deviation from all SDN sections was taken as a threshold. Subsequently, in all sections pixels with gray values above threshold were measured relative to the total ROI and displayed. Following this experiment, we selected sections at positions relative to bregma ranging from anterioposterior (AP) −1.46 to −2.3 mm for quantification of the effect of short term running, resulting in two to three sections per staining and mouse. All sections of the SDN, 2DR, 7DR groups were analyzed accordingly and sections per mouse were averaged.

Proliferating cells in the dentate gyrus were marked with Ki-67 (all phases of the cell cycle except G_0_ and early G_1_; Scholzen and Gerdes, 2000) and BrdU (S-phase; Nowakowski et al., 1989) and counted with the ImageJ cell counter plugin followed by normalization to the area of the dentate gyrus.

### Data analysis

Statistical analysis was performed with GraphPad Prism version 4 (GraphPad Software, San Diego, CA, USA). Data are shown as mean and standard error of the mean (mean ± SEM) unless otherwise stated. For statistical analysis a one-way ANOVA was followed by a Newman-Keuls *post hoc* test (significance thresholds ^*^p < 0.05, ^**^p < 0.01, ^***^p < 0.001). For comparison of DCX expression along the septotemporal axis a linear regression analysis was performed as integrated in GraphPad Prism.

## Results

### Running data

To investigate the early phase of running-induced neurogenesis, mice were randomly assigned in a sedentary control group (N_SDN_ = 12) and two groups with voluntary access to a running wheel for 2 (N_2DR_ = 14) or 7 days (N_7DR_ = 18). To reduce stress for the socially living animals, mice were housed in pairs in cages with or without running wheels. Taking into account that mice were active mainly during the light-off period (7 p.m. to 7 a.m.), we recorded running distance and duration for this period. For analysis, we divided the running distance and time by two which slightly underestimates the values for each mouse since occasionally mice were running together.

**Figure 1:**
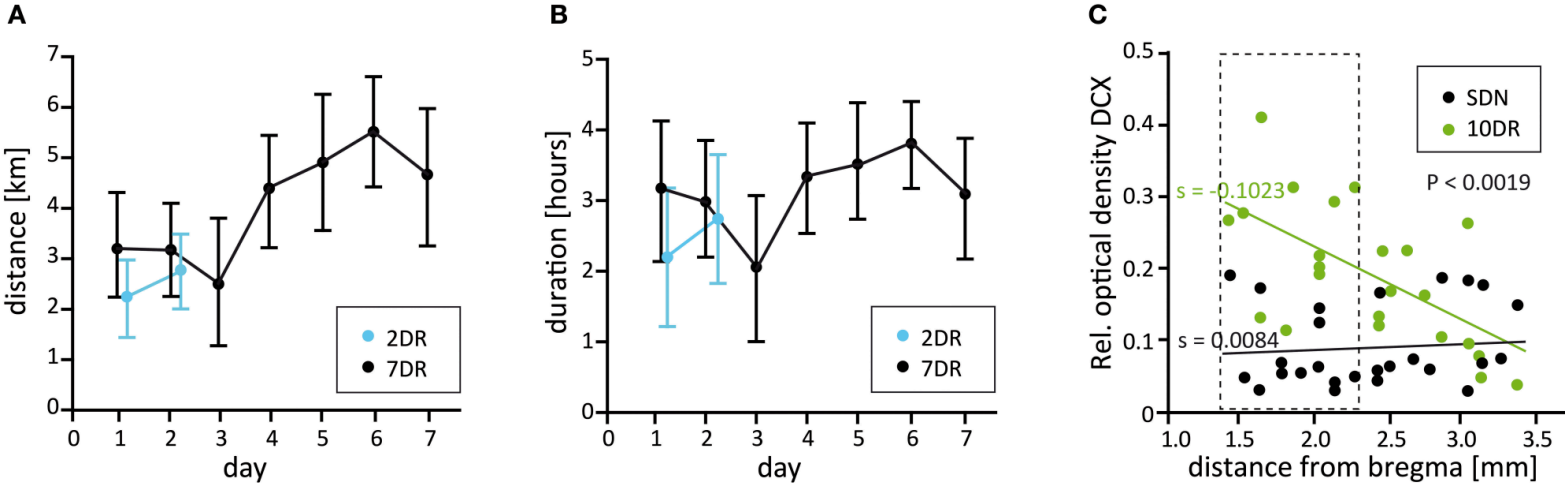
Running parameters and analysis of neurogenesis along the septotemporal axis. **(A)** Mean daily running distance of the 2 day (2DR, blue, *N* = 14) and the 7 day running groups (7DR, black, *N* = 18). Values are given as mean ± 95% confidence interval (CI). **(B)** Mean daily running duration for the 2DR (blue) and 7DR groups (black, mean ± 95% CI). **(C)** Densitometric analysis of DCX immunolabeling along the septotemporal axis for sedentary (SDN) and a preliminary 10 day running group (10DR, green, *N* = 4 mice each) with respect to distance from bregma. Linear regression analysis reveals a significant increase of neurogenesis in the 10DR group (slopes SDN s = 0.0084, 10DR s = −0.1023, *p* < 0.0019). Since the increase of newly born neurons was strongest in the dorsal hippocampus the area between 1.46 and 2.3 mm behind bregma was chosen for further analyses (dashed box).

The 2DR group covered a mean total distance of 4.95 ± 0.27 km (mean ± SEM, range 3.32–6.34 km) within a period of 4.70 ± 0.40 h (range 3.76–6.77 h, daily distance and duration are given in Figures 1A,B). The 7DR group covered a mean total distance of 28.36 ± 3.30 km (range 10.79–54.60 km) within a period of 21.98 ± 2.10 h (range 13.03–38.05 h; daily distance and duration in Figures 1A,B).

### Very short periods lead to alterations in proliferation and neurogenesis

As a prerequisite for our study, we determined whether neurogenesis is differentially affected by physical exercise along the septotemporal axis of the hippocampus. To this end, mice were allowed to use running wheels for 10 days followed by DCX immunolabeling. We found that there is a dorsoventral gradient with the strongest effect of running on the density of DCX-positive cells in the dorsal hippocampus from −1.46 mm to −2.3 mm behind bregma (according to Franklin and Paxinos, 2008; *n* = 4 for runners and SDN, respectively, Figure 1C). Accordingly, we selected this area for further analysis.

To test whether short running periods have an impact on progenitor proliferation and neurogenesis, we performed immunocytochemistry for the proliferation markers Ki-67 and BrdU and for DCX, an established marker for immature granule cells in the SGZ (Brown et al., 2003).

**Figure 2:**
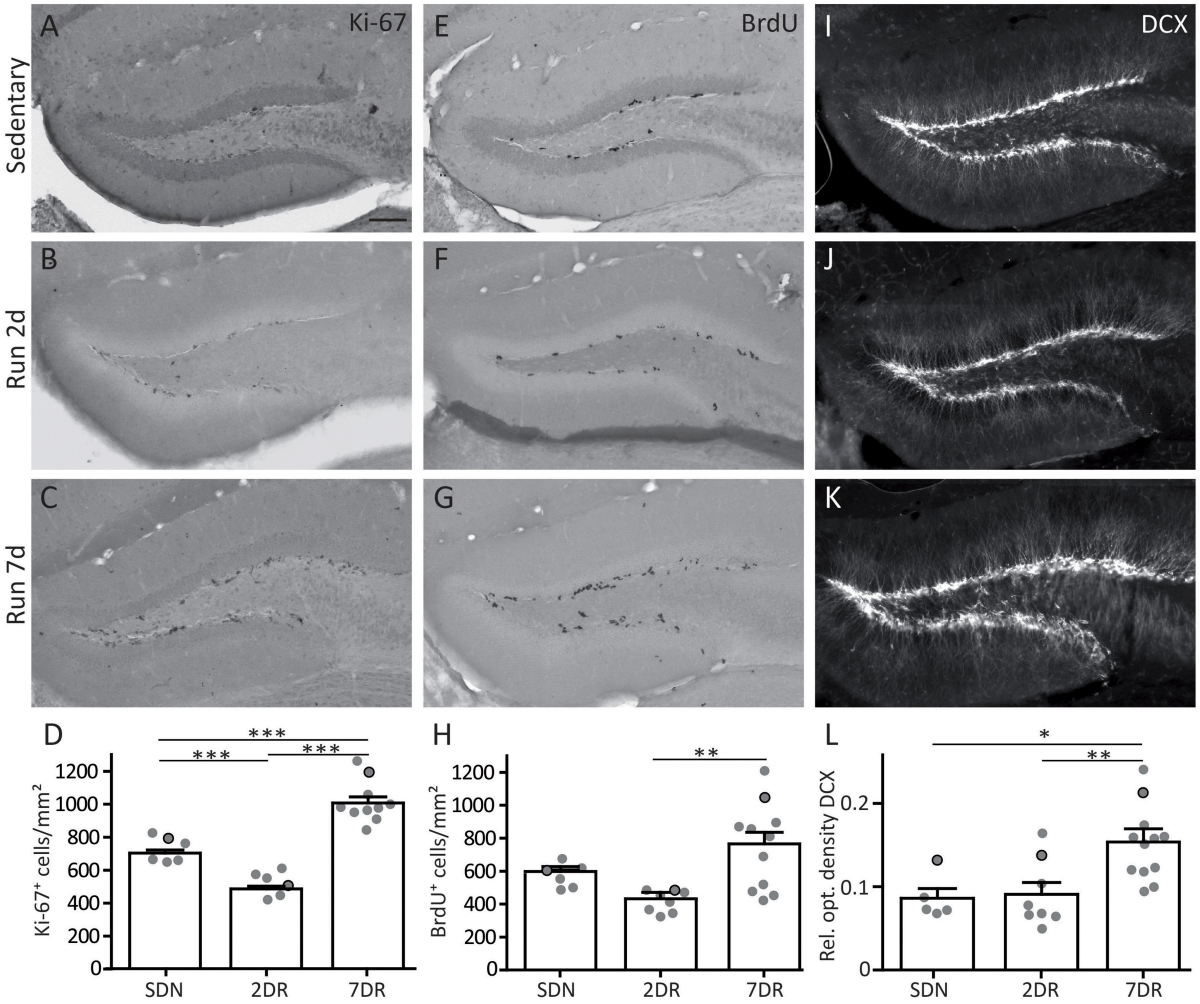
Increased neurogenesis and cell proliferation after 7 days of running is preceded by a transient decrease in proliferation. **(A–C)** Immunocytochemistry for the proliferation marker Ki-67 in sedentary mice (SDN, **A**), and in the 2 day (2DR, **B**) and 7 day running groups (7DR, **C**). Arrows mark single examples of Ki-67-positive cells. **(D)** Quantification of Ki-67-positive cells per area in the dentate gyrus, values are displayed as mean ± standard error of the mean (SEM). The number of proliferating cells was significantly decreased in the 2DR group compared to SDN mice (SDN *N* = 6, 2DR *N* = 7, ANOVA with Newman-Keuls post-test *p* < 0.001). In contrast, in the 7DR group the number of Ki-67-positive cells was significantly increased compared to both other groups (7DR *N* = 10, ANOVA with Newman-Keuls post-test *p* < 0.001 vs. SDN and 2DR). Gray dots with black frame mark the quantitative results for the mice which were selected for the immunostaining in **(A–C)**. All immunostainings in each group stem from the same mouse, respectively. **(E–G)** Immunocytochemistry for the proliferation marker BrdU in the SDN **(E)**, 2DR **(F)**, and 7DR group **(G)**. Arrows mark single examples of BrdU-positive cells **(H)** Quantification of BrdU-positive cells per area in the dentate gyrus. The pattern is comparable to Ki-67-positive cells but significance was only reached for the 7DR vs. the 2DR group (SDN *N* = 6, 2DR *N* = 8, 7DR *N* = 11, ANOVA with Newman-Keuls post-test 7DR vs. 2DR *p* < 0.01; **I–K**). Immunocytochemistry for DCX in the SDN **(I)**, 2DR **(J)**, and 7DR groups **(K)**. Note a salient increase in DCX labeling after 7 days. **(L)** Densitometric analysis of DCX immunolabeling in the subgranular zone, granule cell layer and molecular layer, but sparing the hilus. DCX optical density is comparable in the SDN (*N* = 5) and 2DR groups (*N* = 8) but significantly increased in the 7DR group (*N* = 12, *p* < 0.05 vs. SDN; *p* < 0.01 vs. 2DR; ANOVA with Newman-Keuls post-test). Scale bar 100 μm.

Ki-67 is expressed by proliferating cells in all cell cycle phases except for G_0_ and early G_1_. In controls, a few scattered Ki-67-positive cells were visible in the SGZ (Figure 2A). Interestingly, after 2 days of running cell counting revealed significantly lower numbers of Ki-67-positive cells indicating a decrease in proliferation when compared to controls (Figures 2B,D; SDN: 718.5 ± 30.5 cells/mm^2^, N_SDN_ = 6; 2DR: 497.4 ± 28.9 cells/mm^2^, N_2DR_ = 7, ANOVA *p* < 0.0001, Newman-Keuls post-test *p* < 0.001). In contrast, after 7 days of running a strong increase of Ki-67-positive cells, which were often allocated in clusters, was visible (Figure 2C) and confirmed by cell counting (Figure 2D; 7DR: 1018.0 ± 40.6 cells/mm^2^, N_7DR_ = 10, Newman-Keuls post-test *p* < 0.001 vs. SDN and 2DR).

To substantiate the results obtained by Ki-67 immunostaining, BrdU was injected in the evening of the last running night (day 1 for the 2DR group, day 6 for the 7DR group) and mice were sacrificed the next morning. As a thymidine analog, BrdU integrates into the DNA during the S-phase of the cell cycle (Nowakowski et al., 1989). After 2 days of running BrdU-positive cells showed a scattered pattern comparable to controls (Figures 2E,F) with an on average reduced density of proliferating cells in the 2DR group which yet did not reach significance (Figure 2H; SDN: 586.1 ± 30.3 cells/mm^2^, N_SDN_ = 6; 2DR: 427.4 ± 24.6 cells/mm^2^, N_2DR_ = 8, ANOVA *p* = 0.0055, Newman-Keuls post-test not significant). After 7 days, the number of BrdU-positive cells was strongly increased in the SGZ (Figures 2G,H; 7DR: 747.7 ± 81.5 cells/mm^2^, N_7DR_ = 11, Newman-Keuls post-test *p* < 0.01 vs. 2DR). Thus, the increased proliferation observed after 7 days of running seems to be preceeded by transiently reduced proliferative activity in the SGZ.

To determine whether altered proliferation translates to changes in neurogenesis, we performed immunocytochemistry for DCX. The SDN group showed a dense, approximately one cell layer thick band of DCX-positive young granule cells along the SGZ (Figure 2I). After 2 days of running, the DCX expression pattern was comparable to controls (Figure 2J), although more ramified dendrites were visible in a subset of mice (2/8 mice). After 7 days of running a strong increase in DCX labeling was detectable in the SGZ, the DCX-positive layer was several cell bodies thick and dendrites were more ramified in most mice (9/12 mice; Figure 2K). Densitometric analysis of DCX immunolabeling confirmed that the SDN and the 2DR group were comparable, whereas DCX expression was significantly increased in the 7DR group (Figure 2L; SDN: 0.086 ± 0.011, N_SDN_ = 5, 2DR: 0.091 ± 0.014, N_2DR_ = 8, 7DR: 0.153 ± 0.012, *N* = 12_7DR_; ANOVA *p* = 0.003, Newman-Keuls post-test: SDN vs. 7DR *p* < 0.05; 2DR vs. 7DR *p* < 0.01).

**Figure 3:**
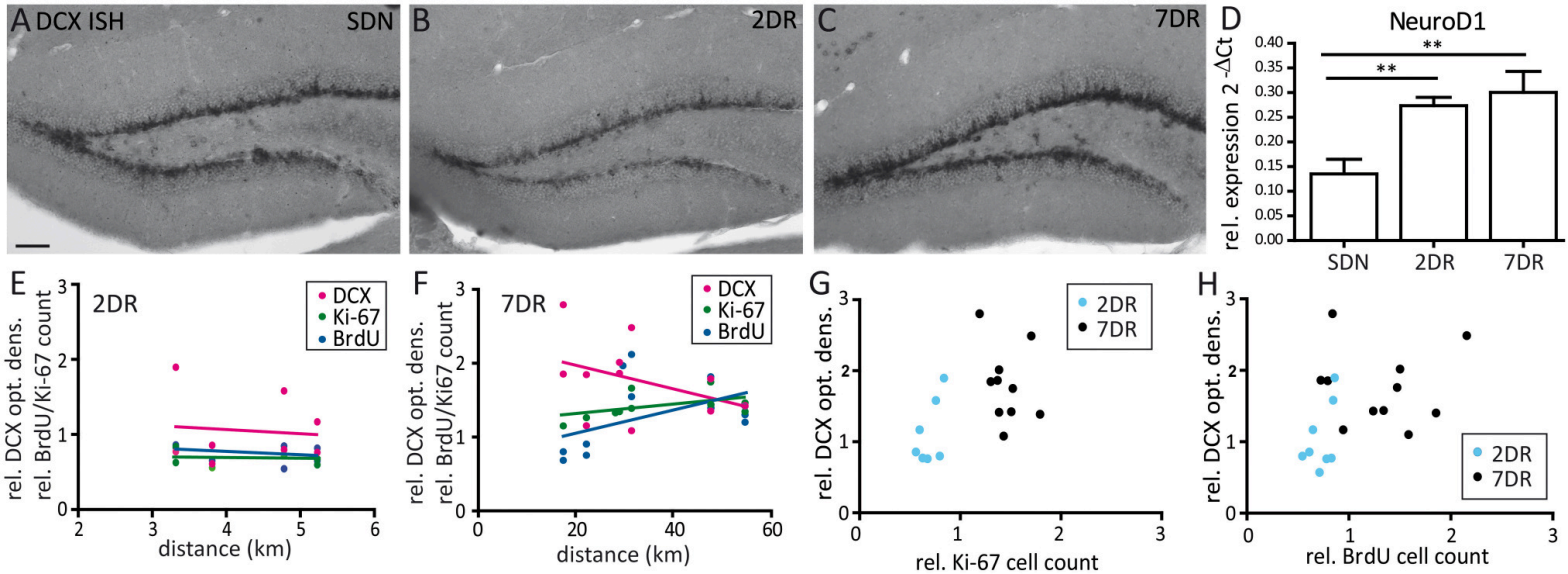
Expression of Ki-67, BrdU, and DCX do not correlate with the running distance. **(A–C)** Representative in situ hybridization for DCX mRNA in an SDN **(A)**, 2DR **(B)**, and 7DR mouse **(C)**. DCX mRNA expression is increased after 7 days of running. **(D)** RT-qPCR analysis for NeuroD1 shows a continuous increase for 2DR and 7DR which was significant compared to the SDN group (*p* < 0.01 for 2DR vs. SDN and 7DR vs. SDN, respectively; ANOVA with Newman-Keuls post-test). RT-qPCR analysis given as 2^−ΔCt^ values relative to the house-keeping gene s12 (*N* = 6 mice per group, values given as mean ± SEM). **(E)** Correlation analysis between running distance and cell counts for BrdU or Ki-67 or optical density of DCX expression in the 2DR group relative to the SDN group. Linear regression analysis revealed no significant correlation for any marker (magenta: DCX, green: Ki-67, blue: BrdU). **(F)** Same as in **(E)** but for the 7DR group. No significant correlation was found between marker expression and running distance. **(G)** Correlation analysis between the relative Ki-67 cell count and optical density of DCX for the 2DR (blue) and 7DR groups (black). There was no significant correlation between both measures within each group. **(H)** Same representation as in **(G)** but for BrdU and DCX. No correlation was found within each group.

To complement these results on the mRNA level, we performed ISH for DCX mRNA which showed comparable expression for the SDN and 2DR group and increased mRNA expression in the 7DR group (Figures 3A–C). In addition, we quantified NeuroD1 mRNA expression by RT-qPCR for NeuroD1 mRNA, another marker for neuronal precursors and young neurons, since RT-qPCR for DCX did not give reliable results in our hands. NeuroD1 mRNA levels were significantly increased already after 2 days of running (Figure 3D; SDN 0.1353 ± 0.0295, 2DR 0.2735 ± 0.01699, 7DR 0.3005 ± 0.0427, *N* = 6 mice per group; one-way ANOVA *p* = 0.0046, Newman-Keuls post-test SDN vs. 2DR *p* < 0.01, SDN vs. 7DR *p* < 0.01) indicating that the increase in neurogenesis after short running periods can be detected even earlier on the mRNA level.

We did not find any significant correlations between the running distance and the marker expression relative to controls, neither for the 2DR group (Figure 3E), nor for the 7DR group (Figure 3F). Furthermore, there was no correlation between the number of proliferating cells and DCX expression within each running group, respectively (Figures 3G,H).

### Time course of neurogenic niche factors after short running periods

The expression patterns of neurogenic niche factors are a mirror of molecular processes happening in the neurogenic niche before being visible on the cellular level. To characterize these during the early phase of running-induced differentiation and proliferation, we performed RT-qPCR to quantify mRNA expression of BDNF, FGF-2, BMP4, and Noggin.

**Figure 4:**
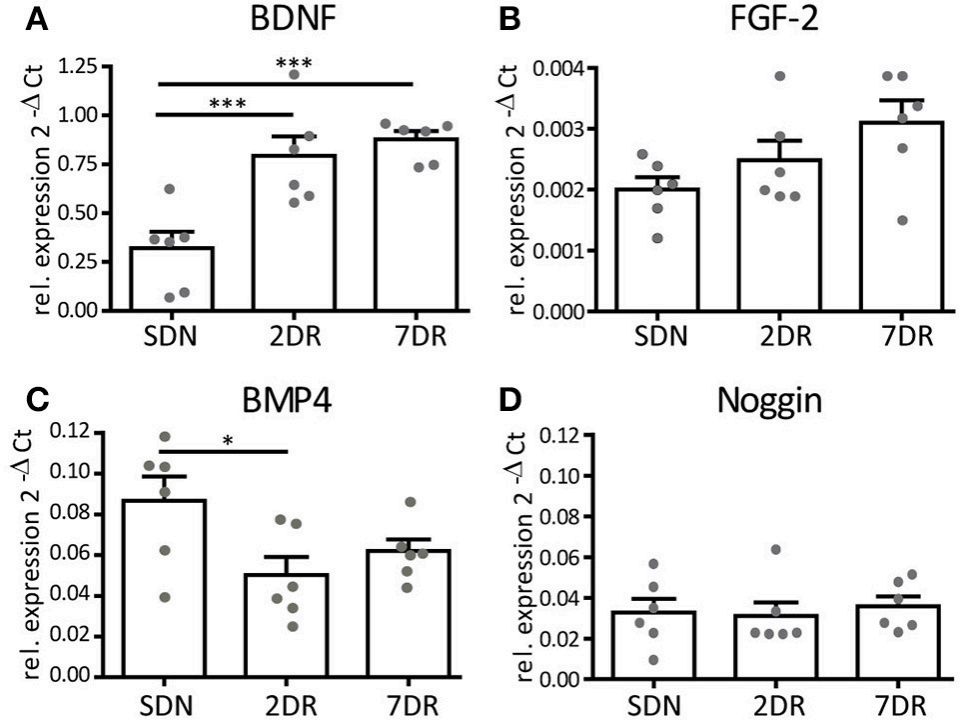
Neurogenic factors show a differential expression profile after short running periods. RT-qPCR analysis for neurogenic factors given as 2^−ΔCt^ values relative to the house-keeping gene s12 (*N* = 6 mice per group, values given as mean ± SEM). **(A)** RT-qPCR analysis for BDNF reveals an early increase in BDNF mRNA expression in the 2DR group that persists in the 7DR group (*p* < 0.001 2DR vs. SDN and 7DR vs. SDN, respectively). **(B)** RT-qPCR analysis for FGF-2 shows a trend toward increasing expression, yet this did not reach significance. **(C)** RT-qPCR for BMP4 shows a decreased expression in the 2DR group compared to the SDN group (*p* < 0.05) but no effect for the 7DR group. **(D)** The expression of the BMP-antagonist Noggin is unaffected by running.

BDNF, which acts as a pro-neurogenic modulator in the neurogenic niche, showed a strong increase in mRNA expression already after 2 days of running (ANOVA, *p* = 0.0003, Newman-Keuls post-test *p* < 0.001 2DR vs. SDN, *n* = 6 each, Figure 4A) and a slight further increase after 7 days (*p* < 0.001 7DR vs. SDN, *n* = 6). The mRNA expression of the pro-neuronal factor FGF-2 showed a trend toward an increase (Figure 4B), yet this failed to reach significance (ANOVA *p* = 0.068, *n* = 6 each).

BMP4 is a modulator of cell proliferation and differentiation, its blockade induces increased proliferation and differentiation (Bonaguidi et al., 2008). We observed a significant reduction of mean BMP4 mRNA expression after 2 days of running (ANOVA *p* = 0.0377, Newman-Keuls post-test *p* < 0.05 2DR vs. SDN, *N* = 6 each; Figure 4C), although single values overlapped between both groups. After 7 days of running, BMP4 mRNA expression was slightly increased yet its mean expression was still below controls. We expected Noggin (the antagonist of BMP) to show opposite values in mRNA expression. However, no differences in Noggin mRNA expression were observed at any time point (ANOVA *p* = 0.85, Figure 4D).

## Discussion

Voluntary wheel running is a well-known, strong stimulator of neurogenesis in the SGZ. In our study, we show that already 2 days of voluntary running are sufficient to induce the neurogenic process as indicated by the upregulation of NeuroD1 mRNA expression—which is reflected on the level of DCX protein and mRNA expression after 7 days. Interestingly, the initiation of differentiation occurs simultaneously with a transient reduction of progenitor cell proliferation, as shown by quantification of Ki-67-positive and BrdU-positive cells after 2 days. We observed that the proliferation rate recovered after 7 days of running and, together with markers for neurogenesis, was even strongly increased by then. Finally, investigation of neurogenic niche factors revealed that BDNF mRNA expression continuously increased during 7 days of running, a comparable, albeit not significant increase was observed for FGF-2. BMP4 mRNA expression showed a different pattern: A significant dip after 2 days was followed by partial recovery after 7 days of running. In contrast, Noggin mRNA expression was unaffected throughout the experiment. We hypothesize that early running-induced neurogenesis is a biphasic process in which differentiation factors first seem to be more potent to stimulate differentiation on the expense of proliferation followed by phase in which proliferation is increased to guarantee an enhanced rate of neurogenesis.

### Time course of wheel usage during 7 days of running

We observed an increase of daily running distance and a slight increase in running time during the course of our study but distances and times were variable across animals and days within each cage pair. This is in agreement with other studies that show variable data during the first up to 10 days when running distances have reached a plateau (Ramsden et al., 2003; Mustroph et al., 2012; Lee et al., 2013; Gregoire et al., 2014).

A dependency of neurogenesis on running duration has been shown: Mice that received access to running wheels for 3 h/day showed higher proliferation rates when compared to only 1 h. This effect was only pronounced if running took place during the active period in the night (Holmes et al., 2004). In addition, a minimum daily distance of 500 m has been indicated to elevate BDNF expression (Cotman and Engesser-Cesar, 2002). With a mean running time of 2–3.5 h at night and a mean distance of 2–5 km our mice were beyond these thresholds guaranteeing sufficient stimulation of neurogenic processes. In fact, it has been shown that intermittent running (3 days/week) has comparable results on neurogenesis as continuous running (7 days/week; Gregoire et al., 2014) which suggests that the variability in running within each pair in our study should not be critical as long as mean running is sufficient. Yet, it has to be kept in mind that it is not fully understood how running time and distance affect neurogenesis (Overall et al., 2016) making interpretation of proliferation data difficult on this basis.

### Neurogenesis is stimulated by short running periods

The majority of studies on running-induced neurogenesis investigated long running periods (>14 days) since their focus was to induce strongly increased neurogenesis to determine underlying mechanisms or the functional effect on neuronal activity and behavioral performance. Only a few studies aimed at clarifying onset processes of running-induced neurogenesis. We show that already after 2 days of voluntary wheel running an increase in the mRNA expression of the transcription factor NeuroD1 occurs in C57Bl/6 mice. On the cellular level, we observed a strong increase in DCX protein expression in somata and dendritic processes after 7 days of running which was not yet detectable after 2 days except for a higher dendritic complexity in a subset of mice.

Comparable results have been found in another study using the same mouse strain: The total number of DCX-positive cells in the whole hippocampus was unchanged after 3 days but strongly increased after 10 days of running (Kronenberg et al., 2006). Interestingly, calretinin which is expressed in young granule cells at a later developmental stage than DCX, was already significantly increased after 3 days (Kronenberg et al., 2006), indicating that the short running period particularly stimulated differentiation. In rats, the number of NeuroD1-expressing cells was shown to be unaltered after 3 days, slightly increased after 7 days and reached a significant increase after 14 days of voluntary wheel running (Patten et al., 2013). In contrast, 3 days of forced, moderate treadmill running (40 min/day) already significantly increased the number of DCX-expressing cells in rats, and neurogenesis remained increased up to 15 days (Ferreira et al., 2011), indicating that voluntary and forced running have differential effects on neurogenesis (Chen et al., 2007). It seems that comparisons between mice and rats have to be handled with care due to a different course of neurogenic processes in both species (Snyder et al., 2009).

In summary, our data are in favor of early pressure toward neuronal differentiation starting already after 2 days of running and indicate that the covered distance was sufficient to activate neurogenic processes.

### Proliferation is transiently reduced after short running periods

Interestingly, we observed reduced proliferation after 2 days for BrdU-positive and, more pronouncedly, for Ki-67-expressing cells. This is in contrast to other studies that analyzed proliferative activity after 3 days of running: In the same mouse strain, Kronenberg et al. (2006) observed higher numbers of BrdU-positive cells. In rats, Ki-67-expressing cells were increased after 3 days of voluntary running (Patten et al., 2013) and more BrdU-positive cells were observed following moderate forced running (Ferreira et al., 2011). The increased proliferation after 7 days revealed with both proliferation markers in our study, however, is in agreement with these other studies. Interestingly, a study that investigated proliferation after 1, 3, and 7 days of wheel running in C57Bl/6 mice, showed increased proliferation after 3 and 7 days but a small, yet not significant drop after 1 day (Van der Borght et al., 2009).

We can only speculate on why we found a drop in proliferation after 2 days in contrast to the increase after 3 days reported by others. Importantly, we can rule out that stress induced by handling during the BrdU injection might be responsible since we found a comparable reduction for Ki-67 expression after 2 days without any handling in a preceding experiment (data not shown). Furthermore, mice were housed in pairs to avoid negative effects on proliferation provoked by single-housing induced stress, as described previously (Stranahan et al., 2006). The different studies used various time points for BrdU injection, i.e., before or after the active/running period of the mice which might lead to differential results in BrdU incorporation. Yet, this does not account for Ki-67 immunocytochemistry, which stains all phases of the cell cycle except for G_0_ (Scholzen and Gerdes, 2000) and is determined by the time point of perfusion.

It is conceivable that during the first 2 days the new environment and the complex movement in a running wheel impose stress on the animals to which they have to adapt to physically (Allen et al., 2001) and that might induce a negative pressure on proliferation. In favor of this hypothesis is the fact that we observed a slightly lower running distance at day 3 and following this adaptation, proliferation strongly increased toward day 7. Furthermore, it has been shown that running changes the duration of the cell cycle mainly by reducing the S-phase of the NeuroD1-expressing cell pool (Farioli-Vecchioli et al., 2014) and these processes might require preceding regulatory steps that result in a transient reduction of proliferation. Yet, on the other hand, short running periods (i.e., 5 days) have been shown not to affect cell cycle length and increased proliferation has been assumed to be caused by increasing the population of proliferating cells (Fischer et al., 2014). One might further hypothesize that running is a trigger to integrate more neurons into the network which are first and quickly provided by differentiation. This decreases the pool of actively proliferating stem cells which is then replenished with a delay to increase proliferation and guarantee a further neurogenesis.

### Early effects in expression of neurogenic niche factors favor neurogenic processes

To investigate how short running periods affect the molecular milieu of the neurogenic niche we measured the expression levels of four niche factors. The neurogenic factor BDNF showed a more than 2-fold increase already after 2 days of running which was preserved after 7 days indicating a strong and immediate regulatory effect. This is in agreement with a couple of other studies that also showed an increase after two and seven (Neeper et al., 1995, 1996) or 3 and 7 days in rats (Molteni et al., 2002; Vaynman et al., 2003). BDNF is important for synaptic plasticity and is associated with the positive effects of wheel running on memory (Neeper et al., 1996; Cotman and Engesser-Cesar, 2002; Vaynman et al., 2003) which might be due to increased neurogenesis and/or to direct effects of BDNF on synaptic performance (Neeper et al., 1995). In our case, increased BDNF expression seems to be—together with increased NeuroD1 mRNA expression—an early indicator for a high pressure toward neuronal differentiation induced by very short running periods.

FGF-2, which plays a role in neuronal differentiation but not proliferation (Werner et al., 2011) only showed a trend toward higher expression for 2 and 7 days. In rats, a transient increase of FGF-2 mRNA expression after 4 days of running has been observed which was not visible after 2 days and had declined after 7 days (Gomez-Pinilla et al., 1997). It is likely that FGF-2 *per se* is not sufficient for the neurogenic effects but requires the interaction with other neurotrophic factors.

BMP signaling is responsible for keeping the stem cell pool quiescent and its inhibition pushes progenitors in an activated proliferative state (Mira et al., 2010; Bond et al., 2014). In our study BMP4 mRNA expression showed a slight reduction after 2 days of running. This is in agreement with another study that found a 50% decrease of BMP4 mRNA also after 2 days in mice (Gobeske et al., 2009), yet in that study proliferation and neurogenesis were not investigated at that time point. The decrease of BMP4 mRNA expression in our study then vanishes slowly and BMP4 mRNA expression after 7 days was no longer significantly different. The regulation of BMP4 is in favor of an early activation of proliferation which is not reflected in our analysis of proliferating cells. Yet, comparable to NeuroD1 and BDNF expression, the reduction of BMP4 might also be an early indicator on mRNA level predicting the increased proliferation that we observed after 7 days. In any case, the result that BMP4 is not as strongly affected as in other studies points toward an involvement of further regulative factors which remain to be determined. It has also been kept in mind in this respect that we cannot predict changes happening on the protein level from our RT-qPCR analysis. Interestingly, the BMP4 antagonist Noggin was unaffected in our study at all-time points, suggesting a Noggin-independent modulation of BMP4. Gobeske et al. (2009) observed a significant increase in Noggin mRNA expression only after 7 days—this is in contrast to our result—but also indicates that BMP4 regulation is taking place earlier and not solely mediated by Noggin.

In summary, the combined expression of neurogenic niche factors after 2 days of running indicates a fast onset and shows that this short period is sufficient to activate the relevant neurogenic processes. In addition, it indicates a high pressure toward differentiation (increased BDNF and NeuroD1 mRNA and – albeit less – FGF-2 mRNA) and toward recruitment or activation of quiescent stem cells to the actively proliferating population (decreased BMP4 expression). Differentiation is then followed by the slightly delayed BMP4 reduction-induced proliferation to fill up the progenitor pool and guarantee further neurogenesis.

## Author contributions

Planned the study and supervised experiments: UH, CH, and JG. Performed experiments: TG, AH, and UH. Analyzed data: TG and UH. Wrote the paper: UH. Commented on the manuscript: CH and JG.

### Conflict of interest statement

The authors declare that the research was conducted in the absence of any commercial or financial relationships that could be construed as a potential conflict of interest.
